# Targeting Glypican-3 in Liver Cancer: Groundbreaking Preclinical and Clinical Insights

**DOI:** 10.3390/biomedicines13071570

**Published:** 2025-06-26

**Authors:** Luca Filippi, Viviana Frantellizzi, Luca Urso, Giuseppe De Vincentis, Nicoletta Urbano

**Affiliations:** 1 Department of Biomedicine and Prevention, University of Rome “Tor Vergata”, 00133 Rome, Italy; 2 Department of Radiological Sciences, Oncology and Anatomical Pathology, “Sapienza” University of Rome, 00161 Rome, Italy; viviana.frantellizzi@uniroma1.it (V.F.); giuseppe.devincentis@uniroma1.it (G.D.V.); 3 Department of Translational Medicine, University of Ferrara, Via Aldo Moro 8, 44124 Ferrara, Italy; luca.urso@unife.it; 4 Nuclear Medicine Unit, Onco-Hematology Department, University Hospital of Ferrara, 44124 Ferrara, Italy; 5 Nuclear Medicine Unit, Department of Onco-Hematology, Fondazione PTV Policlinico Tor Vergata University Hospital, 00133 Rome, Italy; nicoletta.urbano@ptvonline.it

**Keywords:** molecular imaging, theranostics, glypican-3, PET/CT, targeted therapy, oncology

## Abstract

Positron emission tomography (PET) imaging targeting glypican-3 (GPC3) holds promise for improving the detection and characterization of hepatocellular carcinoma (HCC). Preclinical and early clinical studies have largely utilized high-molecular-weight antibodies radiolabeled with isotopes such as ^89^Zr and ^124^I, demonstrating high affinity and tumor uptake but suffering from prolonged circulation times and suboptimal signal-to-background ratios. To address these limitations, interest has shifted toward low-molecular-weight vectors—synthetic peptides and small antibody fragments—labeled with shorter-lived radionuclides (e.g., ^68^Ga and ^18^F) to enable rapid pharmacokinetics and same-day imaging protocols. Emerging platforms such as affibodies and aptamers offer further advantages in target affinity and reduced immunogenicity. However, clinical translation requires rigorous validation: larger, histologically confirmed cohorts, head-to-head comparison with CT/MRI, and correlation with hard clinical endpoints. Moreover, leveraging GPC3 expression as a biomarker could guarantee a deeper knowledge of tumor biology—differentiation grade and vascular invasion risk—and guide theranostic strategies. While β-emitters (^90^Y, ^177^Lu) have been explored for GPC3-directed therapy, their efficacy is influenced by oxygenation and cell-cycle status, whereas α-emitters (^225^Ac) may overcome these constraints, albeit with challenges in radionuclide selection and daughter nuclide management. Finally, dual-targeting probes combining GPC3 and prostate-specific membrane antigen (PSMA) have demonstrated superior uptake and retention in murine models, suggesting a versatile approach for future clinical diagnostics and therapy planning.

## 1. Introduction

Hepatocellular carcinoma (HCC) remains one of the deadliest cancers worldwide, ranking as the third leading cause of cancer-related mortality [1]. Because early-stage HCC is typically asymptomatic and conventional surveillance tools—ultrasonography and serum alpha-fetoprotein—suffer from suboptimal sensitivity and specificity, many patients are diagnosed only once the disease has progressed beyond curative intervention [2,3]. This gap in early detection underscores an urgent need for more reliable, noninvasive strategies to identify and monitor HCC.

Glypican-3 (GPC3) is a cell-surface heparan sulfate proteoglycan overexpressed in 70–80% of HCCs yet virtually absent in normal adult liver and benign hepatic lesions [4,5]. Its tumor-restricted distribution and functional role in key oncogenic pathways—such as Wnt/β-catenin, Hedgehog, and fibroblast growth factor signaling—have established GPC3 as a highly promising biomarker for both imaging and therapy [6]. However, accurately determining and quantifying GPC3 expression through tissue biopsy presents well-known challenges: it is invasive, subject to sampling error in multifocal or disseminated disease, and often cannot capture the dynamic heterogeneity of target expression over time.

Molecular imaging modalities like single-photon emission computed tomography (SPECT) and positron emission tomography (PET) provide a powerful, noninvasive alternative for in vivo visualization and quantification of tumor biomarkers [7,8,9]. By leveraging radiolabeled peptides, antibody fragments, or full antibodies, these techniques potentially enable whole-body assessment of GPC3 expression—revealing not only the presence and intensity of targeting but also intratumoral and interlesional variability. Such comprehensive profiling holds promise for refining patient selection, guiding therapeutic dosing, and monitoring the response to GPC3-directed treatments [10].

Preclinical studies have evaluated diverse arrays of GPC3-binding constructs—peptides, full-length antibodies, antibody fragments, and nucleic acid aptamers—labeled with radionuclides suitable for SPECT or PET imaging, as well as near-infrared dyes for optical imaging. Clinically, the most mature data derive from early-phase trials using radiolabeled codrituzumab (GC33), which demonstrated favorable pharmacokinetics and tumor uptake in HCC patients receiving concurrent sorafenib [11]. A growing pipeline of next-generation tracers—including radiolabeled antibody fragments and small-molecule peptides—has shown high-affinity binding and excellent tumor visualization in animal models, setting the stage for future human studies. Although several therapeutic anti-GPC3 antibodies have also entered clinical evaluation, translational efforts focusing specifically on imaging remain at an early yet rapidly advancing stage.

Our aim is to synthesize and critically appraise the current preclinical and clinical evidence supporting GPC3-targeted imaging and therapeutic approaches in HCC, highlighting key achievements, ongoing challenges, and opportunities for future innovation.

## 2. The Role of Glypican-3 in Hepatocellular Carcinoma

**Figure 1:**
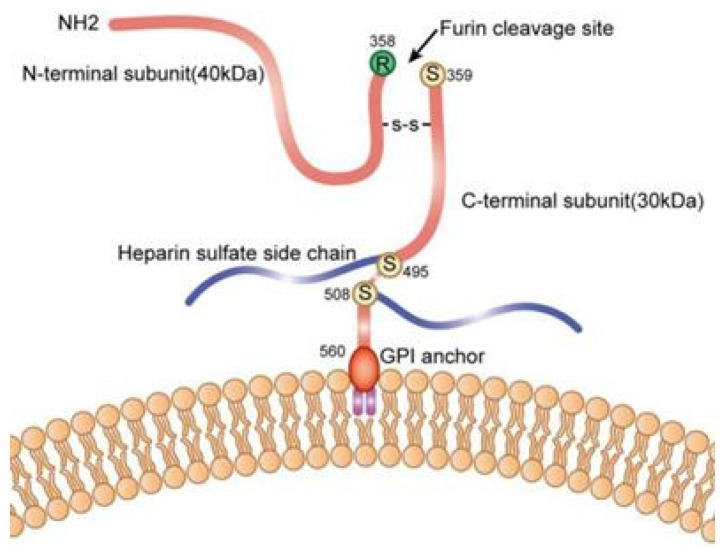
Schematic representation of glypican-3 (GPC3) structure. GPC3 consists of a core protein and a heparan sulfate chain, attached to the cell membrane via a glycosylphosphatidylinositol (GPI) linker. Reprinted from [12], under a creative commons attribution 4.0 international license (http://creativecommons.org/licenses/by/4.0/). No changes were made.

Among the six glypican family members, GPC3 is the most closely associated with liver oncogenesis, showing marked overexpression in HCC while remaining undetectable or minimally expressed in normal adult liver tissue and benign hepatic lesions such as regenerative or dysplastic nodules [12]. GPC3 is cleaved by the furin protease at a specific site (Arg358-Cys359) to form two subunits: a 40 kDa N-terminal subunit and a 30 kDa C-terminal subunit (Figure 1). These subunits are linked by disulfide bonds.

Functionally, GPC3 is involved in several key signaling pathways that drive hepatocarcinogenesis. It facilitates the canonical Wnt/β-catenin signaling pathway by binding to Wnt ligands and their receptors, thereby enhancing downstream transcriptional activation of genes involved in cell proliferation and survival [13]. Additionally, GPC3 modulates the Hippo–YAP signaling axis, with studies demonstrating that GPC3 suppression leads to downregulation of YAP and reduced HCC cell proliferation [14,15]. GPC3 also interacts with the Hedgehog signaling pathway, potentially acting as a competitive inhibitor of Hedgehog ligand binding, thus exerting complex and context-dependent regulatory effects on tumor progression.

Beyond its mechanistic contributions to tumor biology, GPC3 plays a critical role in the clinical landscape of HCC. Immunohistochemical and serum-based studies have confirmed that GPC3 is detectable in a majority of HCC cases, including small or early-stage tumors [16,17]. Notably, GPC3 expression correlates with poor prognosis, aggressive tumor features, and shorter disease-free survival after curative treatment. Its early expression pattern in malignant transformation and minimal expression in non-malignant liver makes GPC3 an ideal candidate for early detection strategies and risk stratification.

Various therapeutic modalities targeting GPC3 have been developed, leveraging its accessibility on the tumor cell surface. Among those monoclonal antibodies (e.g., codrituzumab/GC33), bispecific antibodies, peptide and DNA vaccines, immunotoxins, and chimeric antigen receptor (CAR) T cells can be mentioned, many of which are currently in preclinical or clinical testing [18,19]. Notably, GPC3-targeted CAR-T therapies have demonstrated promising anti-tumor activity in xenograft models and early-phase trials, although challenges related to toxicity and the tumor microenvironment remain [19]. Indeed, the clinical efficacy of GPC3-targeted therapies, particularly CAR-T cells, may be limited by the immunosuppressive tumor microenvironment, which impairs T cell infiltration and persistence, as well as by the risk of severe immune-related adverse events such as cytokine release syndrome and on-target, off-tumor toxicity. Given its restricted expression, biological relevance, and immunogenicity, GPC3 has also gained attention as a potential target for molecular imaging and, eventually, targeted radionuclide therapy.

## 3. Glypican-3-Targeted Imaging

### 3.1. Preclinical Studies

As early as in 2014, Sham et al. [20] proposed the first immuno-PET imaging of HCC. The authors reported the pioneering use of a monoclonal antibody (mAb), labeled with the positron-emitter zirconium-89 (^89^Zr), targeting glypican-3 (^89^Zr-αGPC3). This preclinical study employed orthotopic xenograft models in athymic Nu/J mice using GPC3-positive HepG2 and GPC3-negative RH7777 cells. PET with ^89^Zr-αGPC3 demonstrated high, antigen-specific tumor uptake with excellent tumor-to-liver contrast, even in sub-millimeter lesions. Blocking experiments and heat-denatured controls confirmed target specificity and antibody dependency. In a small-animal PET study, liver and tumor uptake were measured over time in two animals with histologically confirmed tumors of different sizes (3.8 mm vs. <1 mm). The larger tumor showed peak uptake on day 3 (836.6 ± 86.6% ID/g) with a subsequent decline by day 7 (443.9 ± 80.5% ID/g), while liver background activity decreased steadily, resulting in a tumor-to-liver ratio peaking at 32.5 h. In contrast, the smaller tumor exhibited a lower uptake overall, peaking on day 1 (42.5 ± 6.4% ID/g) and dropping to 21.6 ± 3.5% ID/g by day 7. Liver activity in both animals was similar, but the tumor-to-liver ratio in the smaller-tumor animal remained low and stable (1.57–1.4). These findings suggested that GPC3-targeted PET imaging could significantly improve the noninvasive detection and characterization of HCC, paving the way for further investigation.

On the same path, Yang et al. [21] developed and validated an ^89^Zr-DFO-1G12 immuno-PET probe by conjugating ^89^Zr to a GPC3-specific monoclonal antibody (clone 1G12), with the chelator desferrioxamine (DFO). In vitro assays showed high, antigen-dependent uptake and internalization in GPC3-positive HepG2 cells, with minimal binding in GPC3-negative PC3 cells. The in vivo performance of ^89^Zr-DFO-1G12 was evaluated in clinically relevant models of HCC, including orthotopic xenografts from both established HCC cell lines (HepG2 and Hep3B) and primary patient-derived tumors. In cell line-derived xenografts, ^89^Zr-DFO-1G12 showed strong and prolonged uptake in tumors, while background liver signals declined over time, allowing clear tumor delineation by 168 h post-injection. The uptake was highest in HepG2 xenografts and increased over time, whereas Hep3B tumors showed a lower and slightly decreasing uptake, likely reflecting lower GPC3 expression. The tumor-to-liver ratios were high in both models, reaching 6.88 for HepG2 and 5.03 for Hep3B. In xenografts derived from three different HCC patients, ^89^Zr-DFO-1G12 similarly enabled clear tumor visualization starting at 48 h, with continued accumulation in tumors and declining liver uptake over time. Western blot and immunohistochemistry confirmed high GPC3 expression in all patient-derived xenografts. Quantitative analysis revealed high tumor-to-liver ratios across all models (up to 4.21 at 72 h), further supported by biodistribution data. These findings demonstrate the tracer’s strong potential for distinguishing HCC lesions from normal liver tissue across various clinically relevant models.

It has to be noted that mAbs, due to their large size and the intact Fc portion, have some well-known limitations, such as a long circulation time and relatively poor tumor penetration and immunogenicity. A further paper by Sham et al. [22] investigated a novel PET imaging agent, a ^89^Zr-labeled F(ab′)_2_ fragment of an anti-GPC3 antibody (^89^Zr-αGPC3-F(ab′)_2_), designed to overcome limitations of the full mAb. In a preclinical study using orthotopic HCC xenografts in athymic Nu/J mice, the tracer showed rapid blood clearance (half-life ~11 h), strong and specific tumor uptake (peak 100 ± 21% ID/g at 24 h), and superior tumor-to-liver contrast at early time points (T/L ratio of 23.3 at 4 h). Tumors as small as 1.5 mm were clearly visualized on PET as early as 4 h post-injection. Blocking studies confirmed GPC3 specificity, and uptake in GPC3-negative tumors was lower than the background liver signal, minimizing concerns of non-specific binding.

**Figure 2:**
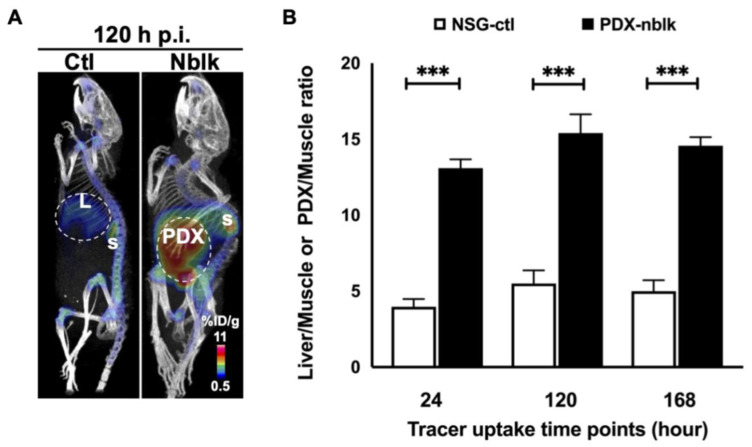
(**A**) PET/CT scans at 120 h post-injection (p.i.) of ^89^Zr-Df-H3K3 show significant uptake in the orthotopic HCC PDX of the non-blocking group (**right side**) compared to the control group (**left side**); L: liver; S: spleen. (**B**) Liver-to-muscle and PDX-to-muscle ratios of ^89^Zr-Df-H3K3 at 24, 120, and 168 h p.i., calculated as the mean ± SD % ID/g (*n* = 4) from ROI analyses of control liver and PDX tissues, *** *p* = 0.001. Reprinted from [23], under a creative commons attribution 4.0 international license (http://creativecommons.org/licenses/by/4.0/). No changes were made.

Natarajan et al. [23] radiolabeled the humanized anti–GPC3 antibody H3K3 with ^89^Zr via a DFO chelator to create ^89^Zr-DFO–H3K3, which selectively binds GPC3-expressing HCC cells. In NSG mice bearing orthotopic, patient-derived HCC xenografts, PET/CT imaging from 4 to 168 h post-injection demonstrated clear tumor delineation against the liver background, with tumor-to-liver ratios exceeding 2.0 as early as 24 h and peaking at 3.4 ± 0.31 by 168 h. Low uptake in non-target tissues and sustained tumor retention suggested that ^89^Zr-DFO–H3K3 might offer a robust contrast for HCC detection, therefore holding promise for clinical translation, potentially enabling same-day imaging or adaptation with shorter-lived isotopes, as shown in Figure 2.

Moving in the same direction, Fayn et al. [24] engineered a human single-domain antibody (HN3) specific for GPC3 and compared two ^89^Zr-DFO PET tracers: a conventional lysine-labeled version (nHN3) and a sortase-mediated site-specifically conjugated version (ssHN3). Both probes retained nanomolar affinity (KD ≃ 10–30 nM) for recombinant GPC3 in vitro, but in mice bearing GPC3-positive A431 or HepG2 xenografts, ^89^Zr-ssHN3 cleared more rapidly from the blood and liver at 1 h post-injection, and ssHN3 achieved a ~7% IA/g tumor uptake versus ~5.7% IA/g for nHN3, yielding a tumor-to-liver ratio of 3.5 ± 0.5 (vs. 1.5 ± 0.5). Kidney uptake was high for both (≈140% IA/g), as expected for sdAbs. Overall, site-specific conjugation markedly improved PET contrast and pharmacokinetics, supporting its further development.

More recently, Dickerson and co-workers [25] assessed the performance of a humanized ^89^Zr-labeled anti-GPC3 antibody (^89^Zr-αGPC3H) versus its murine version (^89^Zr-αGPC3M), employed in the previously cited paper by Sham et al. [20] for immuno-PET imaging in a mouse model of HCC. The underlying motivation was to evaluate the translational suitability of the humanized antibody for clinical use. Both radiotracers showed high radiochemical purity (>98%) and a specific activity of 0.14 GBq/mg. PET imaging demonstrated reliable tumor localization, with the highest tumor-to-liver ratio reaching 24 ± 19% ID/g and tumor uptake up to 170% ID/g, about sevenfold higher than non-target organs. No significant differences were found between the two tracers, supporting further development of ^89^Zr-αGPC3H.

Although ^89^Zr certainly represents an appealing radionuclide for PET imaging, it has several limitations in PET imaging, including its long half-life (78.4 h), which leads to higher radiation exposure for patients and delays optimal imaging time points. In this respect, ^18^F is widely used in PET imaging due to its favorable physical properties, including its short half-life (110 min) and low positron range, which together enable high-resolution images and same-day imaging protocols. Its availability from cyclotrons and versatility in radiochemistry further support broad clinical and research applications.

Wang et al. [26] developed and evaluated a novel ^18^F-labeled peptide-based PET probe, ^18^F-AlF-NODA-MP-6-Aoc-L5, targeting GPC3 in HCC. In this preclinical study, subcutaneous HepG2 xenografts in BALB/c nude mice were used for in vivo imaging. PET/CT performed 60 min post-injection revealed clear tumor visualization with a tumor-to-muscle ratio of 2.46 ± 0.53 but a low tumor-to-liver ratio (0.93 ± 0.16), owing to physiological liver uptake. Blocking experiments with excess peptide confirmed tracer specificity. Although tumor targeting was successful, the authors emphasized the need for further chemical optimization to reduce background liver uptake for effective intrahepatic tumor detection. To address these limitations, the incorporation of a hydrophilic GGGRDN linker into the tetrakaidecapeptide (TP) yielded the novel PET probe Al-^18^F-GP2633, which exhibited markedly reduced hepatobiliary background and enhanced tumor contrast [27]. In HepG2-bearing mice, Al-^18^F-GP2633 achieved a peak tumor-to-liver ratio of 2.00 ± 0.18 at 60 min post-injection, exhibiting a tumor uptake (% ID/g) at 60 min of 3.37 ± 0.35. The hydrophilic modification shifted clearance from hepatic to renal routes, minimizing off-target liver retention. The tracer demonstrated excellent in vitro and in vivo stability (>93% intact at 1 h in tumor and blood). Preclinical micro-PET/CT imaging confirmed GPC3-specific targeting with minimal uptake in GPC3-negative controls.

In a recently published paper, Mo and collaborators reported on a novel fluorinated radiotracer, ^18^F-AlF-NOTA-IPB-GPC3P, for PET imaging GPC3-expressing HCC [28]. This preclinical study was conducted in BALB/c nude mice bearing both subcutaneous and orthotopic Huh7 xenografts. The probe exhibited an excellent tumor uptake (5.05 ± 0.23% ID/g at 120 min post-injection) and favorable tumor-to-background contrast, with tumor-to-muscle and tumor-to-liver ratios reaching 8.15 ± 0.27 and 2.71 ± 0.05, respectively. These findings suggested improved pharmacokinetics and imaging performance compared to previously reported GPC3-targeting agents.

A further area of investigation in GPC3-targeted imaging has been the development of tracers radiolabeled with gallium-68 (^68^Ga). The use of a ^68^Ga-labeled tracer addresses several critical needs in modern nuclear medicine. Thanks to its convenient generator-based production, ^68^Ga provides on-site access to a positron-emitter without reliance on a cyclotron, enabling flexible and cost-effective radiopharmaceutical preparation. Its short half-life (67.7 min) offers a practical balance between allowing complex radiolabeling chemistry and achieving high-quality PET acquisition, while minimizing radiation exposure to patients. Moreover, gallium’s coordination chemistry permits rapid, stable conjugation to targeting vectors—such as peptides, antibodies, or small molecules—facilitating high-contrast imaging of disease biomarkers. In this context, An et al. developed GPC3-targeted immunoPET tracers based on a novel single-domain antibody (“G2”), labeled with both ^68^Ga and ^18^F, for the detection of hepatocellular carcinoma in preclinical mouse models [29]. The ^68^Ga-NOTA-G2 tracer achieved tumor-to-muscle ratios of 4.65 ± 1.12 (Hep3B) and 6.58 ± 0.28 (Huh7) at 1 h post-injection, whereas 18F-G2 reached an even higher ratio of 12.93 ± 3.01 at the same time point. The fusion of G2 to an albumin-binding domain (ABDG2) extended the blood residence time and improved tumor uptake (peak SUV_mean 1.61 ± 0.56 at 6 h), while reducing renal retention compared to the unconjugated sdAb.

### 3.2. First Clinical Applications

The first application of GPC3-targeted imaging in clinical practice was carried out by Carrasquillo’s group [11]. The authors conducted a clinical imaging sub-study within a phase I trial evaluating codrituzumab in combination with sorafenib for advanced HCC. Fourteen patients received a total of 24 injections of codrituzumab, a mAb targeting GPC3, radiolabeled with iodine-124 (^124^I). PET/CT imaging was performed at multiple time points up to 6 days post-injection to assess biodistribution, tumor targeting, and pharmacokinetics. The radiotracer showed tumor uptake in 13/14 patients, with variable intensity (SUVmax > 9 in 6 cases) and peak tumor concentration at 24 h. Imaging revealed limited normal organ accumulation except for thyroid uptake. Repeat scans post-therapy helped to evaluate blocking effects and GPC3 persistence. Dosimetry was computed using VOIs and OLINDA/EXM based on blood and organ kinetics. Dosimetric analysis revealed the thyroid (4.16 ± 2.19 cGy/37 MBq), heart (3.85 ± 0.55), bladder wall (2.73 ± 0.27), and liver (2.43 ± 0.38) as major dose-receiving organs. The effective dose equivalent was 1.65 ± 0.21 cGy/37 MBq for the first study, rising to 2.13 ± 0.36 cGy/37 MBq in patients who underwent a second administration.

A recently published first-in-human study evaluated a synthetic peptide ligand, namely ^68^Ga-RAYZ-8009, for GPC3-targeted imaging in 24 patients (22 adults with suspected or confirmed HCC and 2 children with hepatoblastoma) [30]. Most adult patients (79%) had cirrhosis (various etiologies including alcohol-related and metabolic-associated liver disease), and nearly half had advanced-stage disease (BCLC stage C). PET/CT showed a high tracer uptake in GPC3-positive lesions with rapid clearance from healthy liver, yielding excellent tumor-to-liver ratios (mean TLRmax 8.3; mean TLRmean 7.5 at ~1 h post-injection). Diagnostic performance was assessed semiquantitatively using SUV and TLR metrics, with non-tumor liver as an internal reference. Uptake did not vary significantly with lesion size or LI-RADS classification. The tracer was well tolerated and reliably synthesized, showing potential for the improved detection and staging of HCC, especially in diagnostically indeterminate cases. Table 1 provides a schematic summary of the most relevant findings of GPC3-targeted imaging in preclinical and clinical settings.

## 4. Glypican-3-Targeted Therapy

A preclinical study performed by Ludwig et al. investigated the therapeutic efficacy of the already mentioned GPC3-targeted mAb αGPC3, radiolabeled with the beta-minus-emitter nuclide yttrium-90 (^90^Y) in an HCC model [31]. The antibody was conjugated with ^90^Y using the chelator DOTA (1,4,7,10-Tetraazacyclododecane-1,4,7,10-tetraacetic acid) and administered intravenously to mice bearing orthotopic HepG2-derived HCC xenografts. In vitro, flow cytometry confirmed the preserved binding affinity of the DOTA-αGPC3 conjugate. In vivo, bioluminescence imaging and serum alpha-fetoprotein (AFP) levels—strongly correlated with tumor burden (R^2^ = 0.92)—were used to monitor the response. A single injection of 200 or 300 μCi (e.g., 7.4–11.1 MBq) of ^90^Y-αGPC3 significantly halted or reversed tumor growth, as evidenced by stable or reduced AFP levels and significantly lighter tumor-bearing livers compared to controls. No significant toxicity was observed. While dosimetry per se was not performed, tumor targeting and response assessment via surrogate markers support the potential of GPC3-directed radioimmunotherapy in HCC.

A further investigation from Labadie et al. evaluated a GPC3-targeted theranostic platform for HCC using the same mAb (i.e., αGPC3), conjugated with ^89^Zr (^89^Zr-αGPC3) for PET imaging and ^90^Y (^90^Y-αGPC3) for radioimmunotherapy [32]. In vitro binding was confirmed on HepG2 cells via flow cytometry. In vivo, an orthotopic xenograft mouse model demonstrated effective tumor targeting and volume quantification by immuno-PET, correlating strongly with serum AFP. Treatment with ^90^Y-αGPC3 significantly reduced AFP levels and the gross tumor volume, with increased apoptosis seen on histology. Dosimetry was indirectly assessed via PET-based GTV and serum biomarkers. The approach retained efficacy despite serial antibody exposure and accurately monitored the therapy response.

Bell et al. developed a radioconjugate, ^225^Ac–Macropa–GC33, consisting of the mAb GC33, which targets GPC3, conjugated to the alpha-emitting radionuclide actinium-225 (^225^Ac) through the chelator macropa [33]. This compound was tested in vitro using the HepG2 human liver cancer cell line and in vivo using xenografts in mice. The in vitro studies demonstrated that ^225^Ac–Macropa–GC33 effectively targeted GPC3-expressing cells. In vivo, mice treated with ^225^Ac–Macropa–GC33 exhibited a significant tumor growth delay, particularly in the group receiving 9.25 kBq of the conjugate, suggesting that a lower dose might mitigate toxicity while maintaining therapeutic efficacy. A higher dose of 18.5 kBq, while more effective, caused substantial hematological toxicity, including rapid white blood cell reduction, which is a known side effect of targeted alpha particle therapy (TAT) due to the long circulation times of full-length antibodies. Dosimetry was not detailed in this study, but the results underscored the importance of optimizing dosages and considering strategies like dose fractionation or using smaller targeting ligands to reduce toxicity.

Labadie’s group labeled the antibody αGPC3 with the alpha-emitter thorium-227 (^227^Th) through the chelator p-SCN-Bn-H_4_octapa [34]. In an orthotopic HCC model (i.e., athymic Nu/J mice bearing HepG2-Red-FLuc tumors), bioluminescence imaging tracked tumor growth and revealed significant tumor uptake and retention of the radioimmunoconjugate, with minimal off-target accumulation. Therapeutically, a single dose of ^227^Th-octapa-αGPC3 led to dramatic reductions in tumor burden—confirmed by both imaging and serum α-fetoprotein measurements—and significantly prolonged survival compared to controls. Histopathology and blood work showed acceptable safety and limited damage to healthy tissues. Together, these findings underscore the theranostic potential of GPC3-targeted α-therapy in HCC, combining sensitive tumor imaging with potent tumoricidal alpha irradiation.

In a recent preclinical study, Lin et al. [35] developed and characterized the synthetic peptide RAYZ-8009, conjugated to the chelator DOTA, enabling radiolabeling with either ^177^Lu or ^225^Ac. RAYZ-8009 exhibited a high binding affinity and specificity toward GPC3 across multiple species, including human and mouse, and demonstrated rapid, target-mediated internalization into GPC3-positive HepG2 cells, with up to 58.6% intracellular accumulation within 90 min. In vivo biodistribution studies in nude mice bearing subcutaneous and orthotopic HepG2 xenografts revealed high tumor retention and fast renal clearance, resulting in favorable tumor-to-kidney ratios (peaking at 22.3 at 192 h) and minimal uptake in surrounding healthy tissues. Importantly, treatment with ^177^Lu- or ^225^Ac-labeled RAYZ-8009 induced significant and durable tumor regression with no observable toxicity, and combination with lenvatinib further improved therapeutic efficacy. The sustained intratumoral retention of the radiolabeled peptide supports its utility as a theranostic platform for both the molecular imaging and targeted radiopharmaceutical therapy of GPC3-expressing HCC. Table 2 summarizes the most relevant findings on the various radiolabeled compounds employed for GPC3-targeted therapy. Table 3 provides an overview of the advantages and disadvantages of the different GPC3-targeting molecules employed both for imaging and therapy.

## 5. Discussion

Imaging plays a pivotal role in HCC for early detection, staging, treatment planning, and follow-up. Conventional modalities such as ultrasound, contrast-enhanced computed tomography (ceCT), and magnetic resonance imaging (MRI) primarily assess structural characteristics and vascular patterns, with MRI offering superior sensitivity, particularly for small lesions [36,37,38,39]. However, both ceCT and MRI may fail to detect subcentimeter tumors or early extrahepatic spread. PET/CT, especially with 2-deoxy-2-[^18^F]fluoro-D-glucose (FDG), provides complementary metabolic information, although its diagnostic performance varies depending on tumor differentiation [40]. FDG uptake tends to be higher in poorly differentiated HCC, making PET/CT more valuable in advanced disease, recurrence evaluation, and in cases where conventional imaging yields inconclusive results. Due to the variable sensitivity and limited reliability of FDG PET/CT, particularly in well-differentiated tumors, there is increasing interest in alternative PET tracers, such as ^18^F- or ^11^C-labeled choline and ^11^C-acetate [40]. Nevertheless, these tracers also have important limitations and are metabolic agents, and thus a unsuitable for a theranostic approach. Therefore, there is an unmet need for novel agents specifically designed for HCC-targeted molecular imaging.

The body of evidence presented herein underscores both the promise and the challenges inherent in advancing GPC3-targeted imaging and theranostic strategies for HCC. Preclinical studies have consistently demonstrated that full-length antibodies and engineered fragments can achieve high tumor-to-background contrast, yet their relatively large size and extended circulation times impose limitations on clinical workflow and patient exposure. These observations highlight the necessity of synthesizing and developing lower-molecular-weight agents endowed with faster pharmacokinetics, to ensure imaging windows and signal-to-background ratios that align with the pragmatic demands of clinical care. In this respect, ^68^Ga-labeled peptides have provided a valuable diagnostic platform, leveraging generator-based production for on-site radiochemistry and rapid imaging protocols, which are more suitable for clinical translation than the ^89^Zr-based radiotracers first investigated. However, the inherent characteristics of ^68^Ga-radionuclide—short-range positron emission and a need for germanium–gallium generators—suggest that ^18^F-labeled compounds may ultimately offer superior versatility, enabling centralized cyclotron production, extended distribution networks, and optimal imaging physics for high-resolution, same-day PET studies [41]. Table 4 provides an overview of the various radionuclides employed for GPC3-targeted imaging and therapy.

Beyond radionuclide selection, there is a clear imperative to explore alternative molecular vectors that transcend those already evaluated. Affibodies and aptamers, for instance, exhibit the potential of combining nanomolar affinities for GPC3 with minimal immunogenicity, and their small size promises even more rapid blood clearance and tumor penetration [42,43,44]. Although antibody fragments have bridged the gap between intact immunoglobulins and small peptides, the unique structural properties of these emerging scaffolds warrant systematic investigation, particularly in head-to-head comparisons of in vivo stability, biodistribution, and tumor microenvironment penetration.

On the clinical front, the nascent human data remain limited. To date, only a single trial of a synthetic peptide tracer in 24 patients—of whom not all had histological confirmation—has been reported, leaving the field in need of “hard criteria” rigorous validation. The precedent set by the proPSMA study in prostate cancer demonstrates the feasibility and importance of randomized, prospective imaging trials that benchmark novel PET agents against both cross-sectional modalities (CT and MRI) and gold-standard histopathology [45]. Such rigor is especially pertinent given current EASL (European Association for the Study of the Liver) guidelines, which allow a non-biopsy diagnosis of HCC in high-risk patients (for example, those with cirrhosis or hemochromatosis) based solely on conventional imaging [46]. While this approach is justified by the bleeding risks associated with percutaneous biopsy in coagulopathic patients, it leaves a significant gap in our understanding of the tumor’s underlying biology—its degree of differentiation, proliferative index, and potential for vascular invasion or metastasis. GPC3-targeted imaging has the potential to fill this gap by offering a noninvasive surrogate for tumor aggressiveness, as biomarker expression levels have been correlated with malignancy and metastatic propensity.

In the realm of theranostics and targeted radionuclide therapy, progress has likewise been tempered by reliance on preclinical models and a paucity of robust dosimetric analysis [47,48,49]. In this respect, both ^90^Y and ^177^Lu were investigated for GPC-3 targeted therapy in the preclinical setting. However, these two beta-emitters differ in their tissue penetration and biological behavior. ^90^Y, with its relatively high-energy beta emission and long tissue path length, enhances the crossfire effect and remains advantageous for the treatment of bulky disease [50,51]. However, its therapeutic efficacy is influenced by factors such as tumor oxygenation and the cellular replication cycle. In contrast, ^177^Lu, while emitting lower-energy beta particles with a shorter tissue range, offers distinct advantages when paired with molecular carriers like the GPC3-targeted peptide RAYZ-8009. In HCC cell cultures (HepG2), ^177^Lu-RAYZ-8009 demonstrated rapid and efficient receptor-mediated internalization, with approximately 41.6% of the radioligand internalized within just 20 min, reaching a peak of 58.6% at 90 min [35]. In vivo studies in mice bearing GPC3-positive xenografts confirmed sustained and tumor-specific uptake, accompanied by fast renal clearance and minimal off-target accumulation. This tumor-retentive behavior was further corroborated by clinical imaging data, showing increasing intratumoral uptake for up to 4 h post-injection, while blood and normal tissues showed rapid washout [30]. Such pharmacokinetic and biodistribution characteristics align well with the physical half-life of ^177^Lu (6.7 days), enhancing its therapeutic window and supporting its use in receptor-targeted radiopharmaceutical therapies, particularly for tumors with a high internalization capacity and favorable clearance profiles [52,53]. Indeed, the endocytosis of the GPC3–ligand complex facilitates the intracellular translocation of the radiolabeled agent, positioning the radionuclide in close proximity to the cell nucleus. This intracellular localization is especially critical when employing short-range beta-emitters such as ^177^Lu, whose limited particle path length confines radiation damage to a narrow radius. By concentrating the radionuclide near the nucleus, the probability of inducing double-stranded DNA breaks and achieving effective tumor cell kill is significantly increased. This mechanism enhances the therapeutic precision of ^177^Lu-based radiopharmaceuticals, promoting maximal cytotoxicity within target cells while limiting off-target effects on adjacent healthy tissue.

Alpha-emitters, on the other hand, deliver high-linear energy transfer radiation that is less dependent on microenvironmental factors and can induce lethal double-strand DNA breaks [54,55]. Yet alpha-emitters have limited availability, due to a shortage of production. Moreover, the selection of an appropriate alpha-emitter is complicated by the daughter products that recoil from initial decay and may distribute unpredictably, raising concerns about off-target toxicity. A concerted effort to refine dosimetry, by employing smaller, more rapidly clearing vectors, will be essential to translate this promise into safe, efficacious therapies.

It is also instructive to compare GPC3-targeted approaches with other emerging theranostic platforms, notably those directed against prostate-specific membrane antigen (PSMA). PSMA is overexpressed by the neoendothelium of several solid tumors, including HCC. Early imaging studies using PSMA-ligands have yielded encouraging lesion detection rates [56]. However, since PSMA expression reflects the tumor vasculature rather than the malignant hepatocytes themselves, its capacity as a direct therapeutic target remains uncertain. In contrast, GPC3 is expressed on the tumor cell surface in the majority of HCCs, offering a more direct avenue for both imaging and targeted radionuclide therapy [57]. The comparative evaluation of PSMA and GPC3—each with distinct biological rationales—will be critical in defining the optimal biomarker for HCC theranostics, particularly as we move toward personalized paradigms that integrate imaging phenotypes with molecular and histologic tumor characteristics. In this respect, a dual approach, targeting both PSMA and GPC3, for HCC imaging has recently been investigated as a preclinical study [58]. The dual-imaging probe was synthesized by conjugating TJ12P2, previously a GPC3-targeting peptide, to a highly potent PSMA inhibitor and then complexed with the chelator NOTA (1,4,7-triazacyclononane-1,4,7-triacetic acid). The resulting compound was labeled with both ^18^F and ^68^Ga in murine HCC models and the dual-imaging probe achieved tumor uptakes 30–60% higher than monomeric GPC3 or PSMA tracers, with tumor-to-muscle ratios exceeding 4:1 at 90 min post-injection. Such data suggest that the dual-imaging approach not only benefits from prolonged retention via dual binding but also can adapt to different isotope production workflows. From a clinical perspective, a bispecific tracer could prove most valuable in lesions with heterogeneous marker expression—where GPC3 is low in some niches but PSMA remains high in neovasculature, or vice versa—thus maximizing detection sensitivity across diverse tumor phenotypes.

Although several GPC3-targeted probes and therapies have progressed to early clinical evaluation, the evidence remains limited by small cohort sizes, single-center or case series designs, and short follow-up durations. For imaging modalities, reported studies often involve <20 patients, restricting statistical power for sensitivity/specificity assessments. For therapeutic trials such as CAR-GPC3 T-cell therapy, phase I studies included only a few patients, limiting the ability to detect rare toxicities or robust efficacy signals [19]. Preclinical efficacy in murine xenograft models may not accurately predict human outcomes due to differences in the tumor microenvironment, immune contexture, and GPC3 expression levels between models and patients. Human HCCs are heterogeneous in etiology (e.g., viral vs. nonviral), background liver disease, and molecular subtypes, which may influence probe binding, distribution, and therapeutic responses [59]. Moreover, long-term outcomes (e.g., progression-free survival, overall survival) remain unreported for many early-phase studies, preventing the evaluation of durability.

It is worth noting that, from the perspective of translational applications, a balance between efficacy and safety is paramount in GPC3-targeted therapies. For CAR-T approaches targeting GPC3, early-phase studies report on-target toxicity risks, including cytokine release syndrome (CRS) and potential on-target, off-tumor effects if low-level GPC3 is expressed in normal tissues; close monitoring and step-up dosing strategies may mitigate these risks [60]. For radionuclide-based approaches (e.g., β- or α-particle-labeled anti-GPC3 antibodies), potential hematologic toxicity, liver toxicity, and radiation exposure to adjacent organs must be assessed via dosimetry in preclinical models and early humans [61,62]. Reported preclinical studies show tumor growth inhibition but lack comprehensive toxicity panels; further toxicology studies in larger animals may be warranted. As concerns the employed molecular vectors, antibody–drug conjugates or bispecific formats may carry risks of off-target cytotoxicity and require detailed pharmacokinetic and toxicology profiling [63], while peptide-based therapies generally have lower immunogenicity, but rapid clearance may necessitate repeated administrations, raising cumulative toxicity considerations. These challenges require thorough investigation before GPC-3-targeted therapies can be safely and effectively translated into clinical practice.

An additional avenue worth exploring is the radiolabeling of GPC3-targeting ligands with technetium-99m (^99m^Tc) for SPECT imaging. Although SPECT inherently suffers from lower spatial resolution compared to PET, the widespread availability, lower cost, and robust infrastructure surrounding ^99m^Tc-based diagnostics present a compelling case for its integration into the GPC3 theranostic paradigm [64]. This could be particularly valuable in resource-limited settings, where PET imaging remains inaccessible due to economic or logistical constraints [65]. The ability to perform GPC3-targeted imaging using widely distributed gamma cameras could enable broader early detection, staging, and post-therapy monitoring of hepatocellular carcinoma, especially in regions with a high burden of disease but limited access to advanced nuclear medicine technologies. Developing such agents would extend the reach of molecular imaging beyond high-resource environments, enabling equitable access to precision diagnostics in HCC management.

Looking forward, several priority areas emerge. First, the development of ^18^F-labeled small molecules or peptides targeting GPC3, with streamlined radiochemistry and demonstrably superior image quality, could accelerate clinical translation. Second, the systematic evaluation of novel scaffolds—affibodies, aptamers, and other engineered proteins—will expand the toolkit of vectors available for precise tumor targeting. Third, rigorous, multicenter clinical trials are needed to validate diagnostic performance against histology and conventional imaging, to define the incremental value of GPC3 PET in patient management algorithms, and to establish standardized criteria for image interpretation. Finally, in the theranostic domain, the integration of quantitative imaging biomarkers with refined dosimetric modeling will be paramount to harness the full potential of both beta- and alpha-emitters, ensuring that therapeutic indices are maximized while minimizing toxicity.

## 6. Conclusions

GPC3-directed strategies in HCC represent a rapidly evolving frontier at the intersection of molecular oncology and nuclear medicine. Through iterative optimization of radiochemistry, vector design, clinical validation, and dosimetric rigor, these approaches hold the promise of transforming the early detection, characterization, and treatment of hepatocellular carcinoma.

## Figures and Tables

**Table 1:** Schematic summary of the most relevant findings of GPC3-targeted imaging in the preclinical and clinical settings.

Author	Year	Country	Radiois.	Tracer	Mol. Vector	Setting	Comment	Validation Status
Sham et al. [20]	2014	USA	^89^Zr	^89^Zr-αGPC3	full antibody	Orthotopic HCC xenografts in Nu/J immunodeficient mice	Proof-of-concept study demonstrating the feasibility of immuno-PET targeting of GPC3 in HCC using ^89^Zr-labeled antibodies. The use of an orthotopic model and detailed blocking controls adds translational value to the imaging approach.	Preclinical (mouse xenograft)
Yang et al. [21]	2014	USA	^89^Zr	^89^Zr-DFO-1G12	full antibody	Subcutaneous and orthotopic xenografts in athymic mice	The use of both cell-line and patient-derived orthotopic models, coupled with quantitative PET biodistribution, robustly demonstrates the probe’s specificity and favorable tumor-to-liver contrast.	Preclinical (mouse xenograft)
Sham et al. [22]	2014	USA	^89^Zr	^89^Zr-αGPC3-F(ab′)_2_	Ab fragments	Athymic Nu/J mice with orthotopic xenografts	Immuno-PET with F(ab′)_2_ fragments allowed early high-contrast imaging due to faster clearance and lower background uptake.	Preclinical (mouse xenograft)
Wang et al. [26]	2018	China	^18^F	^18^F-AlF-NODA-MP-6-Aoc-L5 (peptide L5)	synthetic peptide	BALB/c nude mice with subcutaneous HepG2 xenografts	^18^F-labeled PET tracer for imaging GPC3 expression in HCC showed favorable tumor-to-muscle contrast and rapid imaging timeline, though high liver background remains a key limitation.	Preclinical (mouse xenograft)
Li et al. [27]	2020	China	^18^F	Al-^18^F-GP2633	synthetic peptide	HepG2 tumor-bearing nude mice, *n* = 3 per group	The addition of the GGGRDN linker to the TP scaffold effectively improved hydrophilicity and tumor contrast, demonstrating a clear strategy to overcome hepatic clearance issues.	Preclinical (mouse xenograft)
Natarajan et al. [23]	2021	USA	^89^Zr	^89^Zr-DFO-H3K3	humanized antibody	Orthotopic NSG mouse PDX (HCC PDX622)	PET with ^89^Zr-Df-H3K3 allowed high tumor-to-liver ratio (3.4 ± 0.31) at 168 h p.i., achieving specific targeting with minimal background.	Preclinical (mouse xenograft)
An et al. [29]	2022	China	^68^Ga, ^18^F	^68^Ga-NOTA-G2 ^18^F-G2 ^68^Ga-NOTA-ABDG2 (G2 fused to albumin-binding domain)	synthetic peptides	Subcutaneous Hep3B and Huh7 hepatocellular carcinoma xenografts in mice	sdAb-based immuno-PET allowed high-contrast imaging of GPC3-expressing hepatocellular carcinoma, with tumor-to-muscle ratios reaching nearly 13 for the 18F-labeled probe. The fusion to an albumin-binding domain notably improved the pharmacokinetics.	Preclinical (mouse xenograft)
Fayn et al. [24]	2023	USA	^89^Zr	^89^Zr-ssHN3	ssAb portion	Nude mice bearing GPC3-positive HepG2 or A431-GPC3 xenografts	Sortase-based site-specific labeling enhanced the imaging performance of single-domain antibodies in GPC3-expressing liver cancer.	Preclinical (mouse xenograft)
Dickerson et al. [25]	2024	USA	^89^Zr	^89^Zr-αGPC3H vs. ^89^Zr-αGPC3M	humanized antibody	Mouse model of hepatocellular carcinoma	The study compared humanized versus murine 89Zr-αGPC3 antibodies in a mouse model, showing strong tumor targeting and favorable imaging contrast.	Preclinical (mouse model)
Mo et al. [28]	2024	China	^18^F	^18^F-AlF-NOTA-IPB-GPC3P	synthetic peptide	BALB/c nude mice with Huh7 xenografts	GPC3-targeted PET probe with favorable pharmacokinetics, exhibiting high tumor-to-muscle and tumor-to-liver ratios.	Preclinical (mouse xenograft)
Carrasquillo et al. [11]	2018	US	^124^I	^124^I-codrituzumab	full antibody	14 patients with advanced HCC	^124^I-codrituzumab demonstrated high tumor targeting of GPC3-expressing lesions with favorable biodistribution. Dosimetric evaluation showed acceptable organ doses and effective whole-body exposure.	Phase I imaging trial
Poot et al. [30]	2024	the Netherlands and Germany	^68^Ga	^68^Ga-RAYZ-8009	synthetic peptide	24 patients (22 adults with HCC, 2 children with hepatoblastoma)	GPC3-targeted PET imaging outperformed CT/MRI in lesion visibility, particularly in indeterminate LI-RADS categories, also influencing clinical management.	First-in-human (case series)

Radiois.: radioisotope; Mol. Vector: molecular vector; DFO: desferrioxamine; DOTA: dodecane tetraacetic acid; mAb: monoclonal antibody; GPC3: glypican-3; HCC: hepatocellular carcinoma; nota: 1,4,7-triazacyclononane-1,4,7-triacetic acid.

**Table 2:** Summary of the most relevant findings of GPC3-targeted radionuclide therapy in preclinical settings.

Author	Year	Country	Radio.	Tracer	Mol. Vector	Setting	Comment	Validation Status
Ludwig et al. [31]	2019	USA	^90^Y	^90^Y-DOTA-αGPC3	full mAb	HepG2-Red-FLuc cells and orthotopic xenografts	The study offered promising evidence of therapeutic efficacy for ^90^Y-αGPC3 in HCC. Nonetheless, the absence of dosimetric evaluation tempered its immediate clinical applicability.	Preclinical (mouse xenograft)
Labadie et al. [32]	2021	USA	^90^Y ^89^Zr	^89^Zr-DFO-αGPC3 (diagnostic), ^90^Y-DOTA-αGPC3 (therapeutic)	full mAb	HepG2 GPC3+ HCC cells and orthotopic xenograft mouse model of HCC	An integrated theranostic strategy in HCC. The authors investigated the feasibility of an approach combining high target specificity, measurable therapeutic effect and imaging-based monitoring.	Preclinical (mouse xenograft)
Bell et al. [33]	2021	USA	^225^Ac	^225^Ac–Macropa–GC33	full mAb	HepG2 (GPC3+ human hepatoblastoma cell line) and HepG2 subcutaneous xenografts in nude mice	Preclinical efficacy of GPC3-targeted alpha therapy for HCC, though significant hematologic toxicity underscored the need for optimization	Preclinical (mouse xenograft)
Labadie et al. [34]	2022	USA	^227^Th	^227^Th-Bay 2287411	full mAb	Orthotopic xenograft in athymic Nu/J mice using HepG2-Red-FLuc cells	By leveraging a GPC3-specific α-emitter conjugate in an orthotopic HCC model, this study demonstrated an effective theranostic approach—simultaneously enabling sensitive tumor imaging and highly localized, DNA-damaging α-therapy.	Preclinical (mouse xenograft)
Lin et al. [35]	2024	USA	^177^Lu ^225^Ac	^177^Lu-DOTA-RAYZ-8009 ^225^Ac-DOTA-RAYZ-8009	synthetic peptide	HepG2 (GPC3+ HCC) and athymic nude mice with subcutaneous and orthotopic HCC xenografts	The radiolabeled peptide showed favorable pharmacokinetics and biodistribution, demonstrating the potential of RAYZ-8009 as a peptide-based alternative to antibody-based radiopharmaceuticals in HCC theranostics.	Preclinical (mouse xenograft)

Radio.: radioisotope; Mol.: molecular; DFO: desferrioxamine; DOTA: dodecane tetraacetic acid; mAb: monoclonal antibody; GPC3: glypican-3; HCC: hepatocellular carcinoma.

**Table 3:** Comparative overview of GPC3-targeting radiolabeled compounds.

Molecules	MW	Binding Affinity	Circulation/ Half-Life	Tumor Penetration	Clearance Route	Immunogenicity Risk	Production Complexity and Cost	Advantages	Disadvantages	Representative GPC3 Examples and Status
Full-length mAb	~150 kDa	High (nM–pM)	Long (days–weeks)	Moderate–slow	Reticulo-endothelial	Moderate (mitigated by humanization)	High (cell culture; complex purification)	Strong binding; long tumor retention; established manufacturing pipelines	Slow tumor penetration; high background retention in imaging; higher cost; potential immunogenicity if not fully humanized	Humanized anti-GPC3 immunoPET (e.g., 89Zr-labeled): phase I imaging studies in HCC patients; pilot study of 89Zr codrituzumab
Fab/scFv fragment	~25–50 kDa	Moderate–high (nM–sub-nM)	Short (hours)	Faster than full IgG	Renal	Lower than full IgG	Moderate (requires recombinant expression, purification)	Faster blood clearance improving imaging contrast; smaller size aids penetration	Rapid clearance can reduce tumor uptake unless modified; may require engineering for stability or half-life extension	Preclinical scFv-based imaging (mouse xenograft)
Nanobody (sdAb)	~12–15 kDa	Moderate–high (nM–sub-nM)	Very short (hours)	High	Renal	Low	Moderate (microbial expression)	Excellent tissue penetration; rapid imaging contrast; lower immunogenicity risk	Very rapid clearance may necessitate PEGylation or albumin-binding to extend half-life for therapy	Site-specifically conjugated sdAb immuno-PET (preclinical)
Peptide	~1–3 kDa	Moderate (μM–nM)	Very short to short (minutes–hours)	Very high	Renal	Low	Low (chemical synthesis)	Low cost; rapid tumor penetration and fast background clearance for imaging	Lower affinity; rapid degradation/metabolism; may require modifications for stability; short retention limits therapy use	Radiofluorinated GPC3-binding peptides for PET (preclinical); [18F]AlF-NOTA-IPB-GPC3P preclinical evaluation; peptide binder theranostic (preclinical)
Affibody-like scaffold	~6–7 kDa	Engineered high (nM)	Short (hours)	High	Renal	Low	Moderate (recombinant; possible chemical synthesis variants)	Small size with high engineered affinity; good penetration; rapid clearance	Stability may require engineering; limited examples in GPC3 context; rapid clearance may limit therapy unless modified	No published GPC3-specific affibody yet; potential based on affibody use in other targets.
Aptamer	~10–15 kDa (oligonucleotide)	Moderate–high (nM)	Short (minutes–hours)	High	Renal	Very low	Low–moderate (chemical synthesis; modifications needed)	Synthetic production; modifiable conjugation; low immunogenicity	Susceptible to nuclease degradation without modifications; limited stability in vivo; few/no GPC3-specific aptamers published	No published GPC3-targeted aptamer yet; potential based on aptamers’ use in other targets

MW: molecular weight; nM: nanomolar; pM: picomolar; μM: micromolar; kDa: kilodalton.

**Table 4:** Overview of the various radionuclides employed for GPC3-targeted imaging and therapy.

Radionucl.	Application	Emission Type	Energy (MeV)	Half-Life	Pros and Cons
^89^Zr	Imaging	β^+^ (Positron)	0.389 (mean)	78.4 h	Pros: - Matches long antibody kinetics - High image sensitivity Cons: - Prolonged radiation exposure - Delayed imaging window
^124^I	Imaging	β^+^ (Positron)	2.14 (max)	100.8 h	Pros: - Suitable for full antibodies - Extended imaging timeframe Cons: - High-energy positrons reduce resolution - Significant thyroid uptake
^18^F	Imaging	β^+^ (Positron)	0.635 (max)	109.8 min	Pros: - High spatial resolution - Same-day imaging Cons: - Short half-life challenges logistics
^68^Ga	Imaging	β^+^ (Positron)	1.90 (max)	67.7 min	Pros: - Generator-produced on-site - Rapid protocols Cons: - Lower resolution due to higher positron energy - Very short half-life
^90^Y	Therapy	β^−^ (Beta minus)	2.28 (max)	64.1 h	Pros: - Deep tissue penetration - Good for bulky tumors Cons: - Off-target irradiation risk - Influenced by tissue oxygenation
^177^Lu	Imaging Therapy	β^−^ (Beta minus) γ photons	~0.497 (max) 0.208 (11%), 0.113 (6.4%)	6.65 days	Pros: - Well-suited for medium-range tissue penetration (~1–2 mm), ideal for treating small - to medium-sized tumors - Dual capability allows simultaneous therapy and imaging (theranostics) - Established production and clinical track record Cons: - Lower linear energy transfer (LET) compared to α-emitters like ^225^Ac - Potential kidney toxicity
^225^Ac	Therapy	α (Alpha)	~5.9	9.9 days	Pros: - High-linear energy transfer - Effective against resistant cells Cons: - Daughter recoil toxicity - Complex supply and handling
^227^Th	Therapy	α (Alpha)	~5.9	18.7 days	Pros: - High linear energy transfer (LET) - Physical half-life well matched to the slow pharmacokinetics of antibodies Cons: - Complex decay chain - Limited isotope availability

## Data Availability

Data are available in a publicly accessible repository.

## References

[1] Singal A.G., Lampertico P., Nahon P. (2020). Epidemiology and Surveillance for Hepatocellular Carcinoma: New Trends. J. Hepatol..

[2] Quaglia A. (2018). Hepatocellular Carcinoma: A Review of Diagnostic Challenges for the Pathologist. J. Hepatocell. Carcinoma.

[3] Candita G., Rossi S., Cwiklinska K., Fanni S.C., Cioni D., Lencioni R., Neri E. (2023). Imaging Diagnosis of Hepatocellular Carcinoma: A State-of-the-Art Review. Diagnostics.

[4] Filmus J., Selleck S.B. (2001). Glypicans: Proteoglycans with a Surprise. J. Clin. Investig..

[5] De Cat B., David G. (2001). Developmental Roles of the Glypicans. Semin. Cell Dev. Biol..

[6] Zheng X., Liu X., Lei Y., Wang G., Liu M. (2022). Glypican-3: A Novel and Promising Target for the Treatment of Hepatocellular Carcinoma. Front. Oncol..

[7] Israel O., Pellet O., Biassoni L., De Palma D., Estrada-Lobato E., Gnanasegaran G., Kuwert T., La Fougère C., Mariani G., Massalha S. (2019). Two Decades of SPECT/CT—The Coming of Age of a Technology: An Updated Review of Literature Evidence. Eur. J. Nucl. Med. Mol. Imaging.

[8] Filippi L., Schillaci O. (2006). SPECT/CT with a Hybrid Camera: A New Imaging Modality for the Functional Anatomical Mapping of Infections. Expert Rev. Med. Devices.

[9] Duclos V., Iep A., Gomez L., Goldfarb L., Besson F.L. (2021). PET Molecular Imaging: A Holistic Review of Current Practice and Emerging Perspectives for Diagnosis, Therapeutic Evaluation and Prognosis in Clinical Oncology. Int. J. Mol. Sci..

[10] Mossenta M., Busato D., Dal Bo M., Macor P., Toffoli G. (2022). Novel Nanotechnology Approaches to Overcome Drug Resistance in the Treatment of Hepatocellular Carcinoma: Glypican 3 as a Useful Target for Innovative Therapies. Int. J. Mol. Sci..

[11] Carrasquillo J.A., O’Donoghue J.A., Beylergil V., Ruan S., Pandit-Taskar N., Larson S.M., Smith-Jones P.M., Lyashchenko S.K., Ohishi N., Ohtomo T. (2018). I-124 Codrituzumab Imaging and Biodistribution in Patients with Hepatocellular Carcinoma. EJNMMI Res..

[12] Guo M., Zhang H., Zheng J., Liu Y. (2020). Glypican-3: A New Target for Diagnosis and Treatment of Hepatocellular Carcinoma. J. Cancer.

[13] Kolluri A., Ho M. (2019). The Role of Glypican-3 in Regulating Wnt, YAP, and Hedgehog in Liver Cancer. Front. Oncol..

[14] Montalbano M., Rastellini C., McGuire J.T., Prajapati J., Shirafkan A., Vento R., Cicalese L. (2018). Role of Glypican-3 in the Growth, Migration and Invasion of Primary Hepatocytes Isolated from Patients with Hepatocellular Carcinoma. Cell. Oncol. Dordr. Neth..

[15] Piao Q., Bian X., Zhao Q., Sun L. (2025). Unraveling Glypican-3: From Structural to Pathophysiological Roles and Mechanisms—An Integrative Perspective. Cells.

[16] Capurro M., Wanless I.R., Sherman M., Deboer G., Shi W., Miyoshi E., Filmus J. (2003). Glypican-3: A Novel Serum and Histochemical Marker for Hepatocellular Carcinoma. Gastroenterology.

[17] Chen I.-P., Ariizumi S., Nakano M., Yamamoto M. (2014). Positive Glypican-3 Expression in Early Hepatocellular Carcinoma Predicts Recurrence after Hepatectomy. J. Gastroenterol..

[18] Sawada Y., Sakai M., Yoshikawa T., Ofuji K., Nakatsura T. (2012). A Glypican-3-Derived Peptide Vaccine against Hepatocellular Carcinoma. Oncoimmunology.

[19] Shi D., Shi Y., Kaseb A.O., Qi X., Zhang Y., Chi J., Lu Q., Gao H., Jiang H., Wang H. (2020). Chimeric Antigen Receptor-Glypican-3 T-Cell Therapy for Advanced Hepatocellular Carcinoma: Results of Phase I Trials. Clin. Cancer Res. Off. J. Am. Assoc. Cancer Res..

[20] Sham J.G., Kievit F.M., Grierson J.R., Miyaoka R.S., Yeh M.M., Zhang M., Yeung R.S., Minoshima S., Park J.O. (2014). Glypican-3-Targeted 89Zr PET Imaging of Hepatocellular Carcinoma. J. Nucl. Med..

[21] Yang X., Liu H., Sun C.K., Natarajan A., Hu X., Wang X., Allegretta M., Guttmann R.D., Gambhir S.S., Chua M.-S. (2014). Imaging of Hepatocellular Carcinoma Patient-Derived Xenografts Using ^89^Zr-Labeled Anti-Glypican-3 Monoclonal Antibody. Biomaterials.

[22] Sham J.G., Kievit F.M., Grierson J.R., Chiarelli P.A., Miyaoka R.S., Zhang M., Yeung R.S., Minoshima S., Park J.O. (2014). Glypican-3-Targeting F(Ab’)2 for 89Zr PET of Hepatocellular Carcinoma. J. Nucl. Med..

[23] Natarajan A., Zhang H., Ye W., Huttad L., Tan M., Chua M.-S., Gambhir S.S., So S.K. (2021). A Humanized Anti-GPC3 Antibody for Immuno-Positron Emission Tomography Imaging of Orthotopic Mouse Model of Patient-Derived Hepatocellular Carcinoma Xenografts. Cancers.

[24] Fayn S., King A.P., Gutsche N.T., Duan Z., Buffington J., Olkowski C.P., Fu Y., Hong J., Sail D., Baidoo K.E. (2023). Site-Specifically Conjugated Single-Domain Antibody Successfully Identifies Glypican-3-Expressing Liver Cancer by Immuno-PET. J. Nucl. Med..

[25] Dickerson L.K., Lehnert A.L., Hamlin D.K., Labadie K.P., Goodsell K.E., Liu Y., Li Y., Wilbur D.S., Miyaoka R., Park J.O. (2024). Pilot Study of Humanized Glypican-3-Targeted Zirconium-89 Immuno-Positron Emission Tomography for Hepatocellular Carcinoma. EJNMMI Res..

[26] Wang Z., Han Y.-J., Huang S., Wang M., Zhou W.-L., Li H.-S., Wang Q.-S., Wu H.-B. (2018). Imaging the Expression of Glypican-3 in Hepatocellular Carcinoma by PET. Amino Acids.

[27] Li Y., Zhang J., Gu J., Hu K., Huang S., Conti P.S., Wu H., Chen K. (2020). Radiofluorinated GPC3-Binding Peptides for PET Imaging of Hepatocellular Carcinoma. Mol. Imaging Biol..

[28] Mo C., Sun P., Liang H., Chen Z., Wang M., Fu L., Huang S., Tang G. (2024). Synthesis and Preclinical Evaluation of a Novel Probe [18F]AlF-NOTA-IPB-GPC3P for PET Imaging of GPC3 Positive Tumor. Bioorganic Chem..

[29] An S., Zhang D., Zhang Y., Wang C., Shi L., Wei W., Huang G., Liu J. (2022). GPC3-Targeted immunoPET Imaging of Hepatocellular Carcinomas. Eur. J. Nucl. Med. Mol. Imaging.

[30] Poot A.J., Lapa C., Weber W.A., Lam M.G.E.H., Eiber M., Dierks A., Bundschuh R.A., Braat A.J.A.T. (2024). [68Ga]Ga-RAYZ-8009: A Glypican-3-Targeted Diagnostic Radiopharmaceutical for Hepatocellular Carcinoma Molecular Imaging-A First-in-Human Case Series. J. Nucl. Med..

[31] Ludwig A.D., Labadie K.P., Seo Y.D., Hamlin D.K., Nguyen H.M., Mahadev V.M., Yeung R.S., Wilbur D.S., Park J.O. (2019). Yttrium-90-Labeled Anti-Glypican 3 Radioimmunotherapy Halts Tumor Growth in an Orthotopic Xenograft Model of Hepatocellular Carcinoma. J. Oncol..

[32] Labadie K.P., Ludwig A.D., Lehnert A.L., Hamlin D.K., Kenoyer A.L., Sullivan K.M., Daniel S.K., Mihailovic T.N., Sham J.G., Orozco J.J. (2021). Glypican-3 Targeted Delivery of 89Zr and 90Y as a Theranostic Radionuclide Platform for Hepatocellular Carcinoma. Sci. Rep..

[33] Bell M.M., Gutsche N.T., King A.P., Baidoo K.E., Kelada O.J., Choyke P.L., Escorcia F.E. (2020). Glypican-3-Targeted Alpha Particle Therapy for Hepatocellular Carcinoma. Molecules.

[34] Labadie K.P., Hamlin D.K., Kenoyer A., Daniel S.K., Utria A.F., Ludwig A.D., Kenerson H.L., Li L., Sham J.G., Chen D.L. (2022). Glypican-3-Targeted 227Th α-Therapy Reduces Tumor Burden in an Orthotopic Xenograft Murine Model of Hepatocellular Carcinoma. J. Nucl. Med..

[35] Lin F., Clift R., Ehara T., Yanagida H., Horton S., Noncovich A., Guest M., Kim D., Salvador K., Richardson S. (2024). Peptide Binder to Glypican-3 as a Theranostic Agent for Hepatocellular Carcinoma. J. Nucl. Med..

[36] Simpson H.N., McGuire B.M. (2015). Screening and Detection of Hepatocellular Carcinoma. Clin. Liver Dis..

[37] Chen J., Zhu J., Zhang C., Song Y., Huang P. (2020). Contrast-Enhanced Ultrasound for the Characterization of Portal Vein Thrombosis vs. Tumor-in-Vein in HCC Patients: A Systematic Review and Meta-Analysis. Eur. Radiol..

[38] Anis M. (2015). Imaging of Hepatocellular Carcinoma: New Approaches to Diagnosis. Clin. Liver Dis..

[39] Minami Y., Sugimoto K., Kuroda H., Kamiyama N., Ogawa C., Kudo M. (2025). Differentiating between Hepatocellular Carcinoma and Its Mimickers Using Contrast-Enhanced Ultrasound with Perflubutane Microbubbles. Expert Rev. Med. Devices.

[40] Nyakale N., Filippi L., Aldous C., Sathekge M. (2023). Update on PET Radiopharmaceuticals for Imaging Hepatocellular Carcinoma. Cancers.

[41] Sahnoun S., Conen P., Mottaghy F.M. (2020). The Battle on Time, Money and Precision: Da[18F] Id vs. [68Ga]Liath. Eur. J. Nucl. Med. Mol. Imaging.

[42] Tolmachev V., Orlova A. (2020). Affibody Molecules as Targeting Vectors for PET Imaging. Cancers.

[43] Zhang L., Zhang H. (2024). Recent Advances of Affibody Molecules in Biomedical Applications. Bioorg. Med. Chem..

[44] Bohrmann L., Burghardt T., Haynes C., Saatchi K., Häfeli U.O. (2022). Aptamers Used for Molecular Imaging and Theranostics—Recent Developments. Theranostics.

[45] Hofman M.S., Lawrentschuk N., Francis R.J., Tang C., Vela I., Thomas P., Rutherford N., Martin J.M., Frydenberg M., Shakher R. (2020). Prostate-Specific Membrane Antigen PET-CT in Patients with High-Risk Prostate Cancer before Curative-Intent Surgery or Radiotherapy (proPSMA): A Prospective, Randomised, Multicentre Study. Lancet.

[46] European Association for the Study of the Liver (2025). EASL Clinical Practice Guidelines on the Management of Hepatocellular Carcinoma. J. Hepatol..

[47] Tran-Gia J., Cicone F., Koole M., Giammarile F., Gear J., Deshayes E., Gabiña P.M., Cremonesi M., Wadsley J., Bernhardt P. (2025). Rethinking Dosimetry: A European Perspective. J. Nucl. Med..

[48] Burkett B.J., Bartlett D.J., McGarrah P.W., Lewis A.R., Johnson D.R., Berberoğlu K., Pandey M.K., Packard A.T., Halfdanarson T.R., Hruska C.B. (2023). A Review of Theranostics: Perspectives on Emerging Approaches and Clinical Advancements. Radiol. Imaging Cancer.

[49] Sidrak M.M.A., De Feo M.S., Corica F., Gorica J., Conte M., Filippi L., Schillaci O., De Vincentis G., Frantellizzi V. (2023). Fibroblast Activation Protein Inhibitor (FAPI)-Based Theranostics-Where We Are at and Where We Are Heading: A Systematic Review. Int. J. Mol. Sci..

[50] Filippi L., Di Costanzo G.G., Tortora R., Pelle G., Saltarelli A., Marino Marsilia G., Cianni R., Schillaci O., Bagni O. (2020). Prognostic Value of Neutrophil-to-Lymphocyte Ratio and Its Correlation with Fluorine-18-Fluorodeoxyglucose Metabolic Parameters in Intrahepatic Cholangiocarcinoma Submitted to 90Y-Radioembolization. Nucl. Med. Commun..

[51] Cozmin M., Lungu I.I., Cernei R., Marin G.A., Duceac L.D., Calin G., Dabija M.G., Gutu C., Goroftei E.R.B., Stefanache A. (2025). Harnessing Radionuclides: Unveiling the Promising Role of Radiopharmaceuticals in Cancer Theranostics and Palliative Care. Curr. Radiopharm..

[52] Nitipir C., Niculae D., Orlov C., Barbu M.A., Popescu B., Popa A.M., Pantea A.M.S., Stanciu A.E., Galateanu B., Ginghina O. (2017). Update on Radionuclide Therapy in Oncology. Oncol. Lett..

[53] Pavel M., Caplin M.E., Ruszniewski P., Hertelendi M., Krenning E.P., Strosberg J.R. (2025). NETTER-1 Study Group Relationship Between Best Tumor Shrinkage and Progression-Free Survival and Overall Survival in Patients With Progressive Midgut Neuroendocrine Tumors Treated With [177Lu]Lu-DOTA-TATE: Ad Hoc Analysis of the Phase III NETTER-1 Trial. Cancer Med..

[54] King A.P., Lin F.I., Escorcia F.E. (2021). Why Bother with Alpha Particles?. Eur. J. Nucl. Med. Mol. Imaging.

[55] Guerra Liberal F.D.C., O’Sullivan J.M., McMahon S.J., Prise K.M. (2020). Targeted Alpha Therapy: Current Clinical Applications. Cancer Biother. Radiopharm..

[56] Hannah N., Yu C., Nedumannil L., Haridy J., Kong G., Boussioutas A., Sood S. (2024). Prostate-Specific Membrane Antigen (PSMA) PET/CT in the Detection and Diagnosis of Hepatocellular Carcinoma (HCC): A Systematic Review and Meta-Analysis. Cancers.

[57] Filippi L., Braat A.J., Schillaci O. (2022). The Era of Prostate-Specific Membrane Antigen (PSMA)-Based Theranostics for Hepatocellular Carcinoma Is Upcoming: Are We Ready for It?. Eur. J. Nucl. Med. Mol. Imaging.

[58] Chen L., Cheng S., Zhu D., Bao G., Wang Z., Deng X., Liu X., Ma X., Zhao J., Zhu L. (2025). Synthesis and Preclinical Evaluation of Dual-Specific Probe Targeting Glypican-3 and Prostate-Specific Membrane Antigen for Hepatocellular Carcinoma PET Imaging. Mol. Pharm..

[59] Safri F., Nguyen R., Zerehpooshnesfchi S., George J., Qiao L. (2024). Heterogeneity of Hepatocellular Carcinoma: From Mechanisms to Clinical Implications. Cancer Gene Ther..

[60] Chohan K.L., Siegler E.L., Kenderian S.S. (2023). CAR-T Cell Therapy: The Efficacy and Toxicity Balance. Curr. Hematol. Malig. Rep..

[61] Feuerecker B., Kratochwil C., Ahmadzadehfar H., Morgenstern A., Eiber M., Herrmann K., Pomykala K.L. (2023). Clinical Translation of Targeted α-Therapy: An Evolution or a Revolution?. J. Nucl. Med..

[62] Marcu L., Bezak E., Allen B.J. (2018). Global Comparison of Targeted Alpha vs. Targeted Beta Therapy for Cancer: In Vitro, in Vivo and Clinical Trials. Crit. Rev. Oncol. Hematol..

[63] Cho J., Bok H., Jo T., Ahn S. (2025). Analysis of Phase I Clinical Trial Design of Anti-Cancer Agents. Ther. Innov. Regul. Sci..

[64] Emmett L. (2025). SPECT Deserves RESPECT: The Potential of SPECT/CT to Optimize Patient Outcomes with Theranostics Therapy. J. Nucl. Med..

[65] Boschi A., Urso L., Uccelli L., Martini P., Filippi L. (2024). 99mTc-Labeled FAPI Compounds for Cancer and Inflammation: From Radiochemistry to the First Clinical Applications. EJNMMI Radiopharm. Chem..

